# Endothelial Progenitor Cells Dysfunctions and Cardiometabolic Disorders: From Mechanisms to Therapeutic Approaches

**DOI:** 10.3390/ijms22136667

**Published:** 2021-06-22

**Authors:** Anne-Christine Peyter, Jean-Baptiste Armengaud, Estelle Guillot, Catherine Yzydorczyk

**Affiliations:** 1 Neonatal Research Laboratory, Department Woman-Mother-Child, Clinic of Neonatology, Lausanne University Hospital, 1011 Lausanne, Switzerland; Anne-Christine.Peyter@chuv.ch; 2 Faculty of Biology and Medicine, University of Lausanne, 1011 Lausanne, Switzerland; jean-baptiste.armengaud@chuv.ch (J.-B.A.); estelle.guillot@unil.ch (E.G.); 3 Division of Pediatrics, DOHaD Laboratory, Department Woman-Mother-Child, Lausanne University Hospital, 1011 Lausanne, Switzerland

**Keywords:** developmental programming, intrauterine growth restriction, metabolic syndrome, endothelial progenitor cells, oxidative stress, cellular senescence

## Abstract

Metabolic syndrome (MetS) is a cluster of several disorders, such as hypertension, central obesity, dyslipidemia, hyperglycemia, insulin resistance and non-alcoholic fatty liver disease. Despite health policies based on the promotion of physical exercise, the reduction of calorie intake and the consumption of healthy food, there is still a global rise in the incidence and prevalence of MetS in the world. This phenomenon can partly be explained by the fact that adverse events in the perinatal period can increase the susceptibility to develop cardiometabolic diseases in adulthood. Individuals born after intrauterine growth restriction (IUGR) are particularly at risk of developing cardiovascular diseases (CVD) and metabolic disorders later in life. It has been shown that alterations in the structural and functional integrity of the endothelium can lead to the development of cardiometabolic diseases. The endothelial progenitor cells (EPCs) are circulating components of the endothelium playing a major role in vascular homeostasis. An association has been found between the maintenance of endothelial structure and function by EPCs and their ability to differentiate and repair damaged endothelial tissue. In this narrative review, we explore the alterations of EPCs observed in individuals with cardiometabolic disorders, describe some mechanisms related to such dysfunction and propose some therapeutical approaches to reverse the EPCs dysfunction.

## 1. Components of Metabolic Syndrome

The incidence and prevalence of metabolic syndrome (MetS) are increasing worldwide and MetS is becoming a global health problem. MetS affects 20–30% of the population in developed countries [1]. The major components of the MetS cluster are obesity, in particular abdominal body fat accumulation, impaired glucose metabolism, dyslipidemia and arterial hypertension [2,3]. Additionally, non-alcoholic fatty liver disease (NAFLD), which is defined as excess fat (>5% weight or volume) deposition in the liver in the absence of excessive alcohol intake, has been identified as a hepatic manifestation of MetS [4]. NAFLD has emerged as the most common cause of liver disease, and it is associated with substantial morbidity and mortality in Western countries. Moreover, MetS has been identified as an epidemiologic tool related to cardiovascular disease (CVD) risk. In fact, each component of the MetS represents an independent risk factor for the development of CVD [5,6,7].

## 2. Metabolic Syndrome and Endothelial Dysfunction

The endothelium is a thin monocellular layer that covers the inner surface of blood vessels, separating the circulating blood from the interstitial fluid [8], and plays an essential role in the maintenance of vascular homeostasis [9]. Under physiological conditions, the endothelium synthesizes paracrine factors, such as nitric oxide (NO), prostacyclin, endothelium-derived hyperpolarizing factor and natriuretic peptide type-C. These factors regulate the balance between vasodilation and vasoconstriction, inhibit and improve the proliferation and migration of smooth muscle cells, prevent and stimulate the adhesion and aggregation of platelets, and regulate thrombogenesis and fibrinolysis [10,11,12]. NO is the major contributor of these endothelial functions. NO is a gaseous molecule generated during the conversion of L-arginine to L-citrulline via the action of the endothelial nitric oxide synthase (eNOS), which requires the presence of tetrahydrobiopterin (BH_4_) as cofactor [13]. Endothelial function can be explored notably by infusion of acetylcholine, which stimulates endothelial muscarinic receptors, leading to an increase in cytosolic Ca^2+^ and thus to the activation of eNOS to metabolize L-arginine into L-citrulline and NO [14]. Measurement of flow-mediated vasodilation (FMD) by ultrasound allows a noninvasive assessment of endothelial function in patients [15]. Given the large range of the vasoprotective effects of NO, the term “endothelial dysfunction” generally refers to reduced NO bioavailability, notably due to decreased eNOS expression or activity, resulting in enhanced vasoconstrictor responses and thus impaired endothelium-dependent vasodilation [16].

Therefore, an alteration in the structural and functional integrity of the endothelium can lead to the development of cardiometabolic disorders. All components of MetS can individually be associated with impaired endothelial function. Several studies have shown that hypertension [17,18] and abdominal obesity [19,20,21] are associated with endothelial dysfunction. In patients with type-2 diabetes, elevated glucose levels lead to glycosylation of vascular endothelium, resulting in changes in blood vessels such as narrowing and sprouting of neovasculature that is friable and at risk of rupture [22,23,24]. It has been shown that patients with type-2 diabetes have a 2- to 4-fold increased risk of developing CVD compared with non-diabetic individuals [25], related to endothelial dysfunction, as a consequence of inflammation, increased reactive oxygen species (ROS) production and deletion of eNOS [26,27,28]. Patients with NAFLD and non-alcoholic steatohepatitis also display an impaired endothelial function characterized by decreased FMD [29,30,31].

## 3. Endothelial Progenitor Cells

The endothelial progenitor cells (EPCs) are circulating components of the endothelium. They are mobilized and migrate in the circulation from the bone marrow, differentiate into mature endothelial cells, and synthesize and release a wide range of active molecules and growth factors modulating vasculogenesis and improving vascular homeostasis [32,33]. A close association has been identified between the maintenance of endothelial structure and function by EPCs and their ability to differentiate and repair damaged endothelial tissue [34]. EPCs are most frequently isolated from cord blood [35,36] or peripheral blood [35,37], but can also be isolated from pulmonary artery endothelium [38,39] or placenta [40], or can be derived from induced pluripotent stem cells [41]. EPCs can be distinguished according to their phenotype and functional properties in vivo [42]. Early EPCs have a hematopoietic origin and promote angiogenesis through paracrine mechanisms but cannot give rise to mature endothelial cells [42,43,44,45]. In contrast, endothelial colony forming cells (ECFCs) or late outgrowth EPCs [42] have clonal potential and the capabilities to yield mature endothelial cells and to promote vascular formation in vitro and in vivo. In particular, these cells are capable of proliferation, autorenewal, migration, differentiation, vascular growth and neovascularization. It has been demonstrated that ECFCs can be characterized by the assessment of surface markers, such as CD34 and vascular endothelial growth factor receptor-2 (VEGFR-2, also named KDR) [46,47], but the absence of CD45. Importantly, the CD34^+^KDR^+^ combination is the only putative ECFCs phenotype that has been repeatedly and convincingly demonstrated to be an independent predictor of cardiovascular outcomes [48,49]. EPCs have not only a potential therapeutic impact on endothelial dysfunction in both clinical [50] and experimental [51] studies, but also the circulating levels of EPCs can be used as a clinical marker of disease progression [52]. EPCs dysfunction has been characterized by decreased number and/or impaired function of circulating precursors [53]. The number of circulating EPCs was found to be negatively correlated with cardiovascular risk factors and vascular function and to predict CVD independently of both conventional and non-traditional cardiovascular risk factors [54,55,56].

## 4. Endothelial Progenitor Cells Dysfunction in Cardiometabolic Disorders

### 4.1. Endothelial Progenitor Cells Dysfunction in Type-2 Diabetes

In patients with type-2 diabetes, a decrease in the number of EPCs has been reported, and the number of EPCs was lower as more numerous were the complications [57]. Notably a reduction in CD34^+^ EPCs has been mentioned in early stages of type-2 diabetes, which persists thereafter and worsens in patients with diabetes complications [58]. EPCs isolated from patients with type-2 diabetes have demonstrated impaired functions such as alterations of proliferation, migration, chemokinesis, angiogenesis and NO bioavailability [59] compared to nondiabetic patients [60,61,62], as observed also in EPCs from animal models of diabetes [63,64,65,66].

In addition, it is well known that endothelial dysfunction induced by hyperglycemia leads to micro- and macro-angiopathies complications [67]. A reduced number of EPCs in type-2 diabetes has been associated to increased brachial-ankle pulse wave velocity related to arterial stiffness [68] and is correlated to the prevalence of peripheral vascular disease [69,70,71] and with the degree of atherosclerosis [72]. Additionally, high glucose might impair EPCs by modifying NO-related mechanisms [73].

### 4.2. Endothelial Progenitor Cells Dysfunction in Hypertension and Cardiovascular Diseases

EPCs seem to play a protective role against the development of CVD [74]. In fact, several CVD have been associated with altered EPCs number and functions. In prehypertensive patients, an impaired formation of EPCs colonies has been mentioned [75]. Patients with hypertension and vascular lesions displayed a reduced number of circulating CD34^+^ cells [76]. In contrast, Skrzypkowska et al. observed that patients with essential hypertension had increased proportions of CD34^+^ EPCs [77], which may be a compensatory mechanism. Marketou et al. did not find any significant difference in the number of circulating CD34^+^ cells between hypertensive and normotensive individuals, but found a correlation between the number of circulating CD34^+^ cells and pulse wave velocity in hypertensive patients, suggesting a role for EPCs in the pathophysiology of arterial stiffness and arterial remodeling [78]. Hypertension is a risk factor for the incidence of other CVD such as stroke, coronary artery disease, sudden death, heart failure and peripheral arterial disease [79,80]. Hill et al. found a correlation between the number of circulating EPCs and the patient’s combined Framingham risk factor score, which includes six coronary risk factors, such as age, gender, total cholesterol, high density lipoprotein cholesterol, smoking habits and systolic blood pressure value [81,82]. Vasa et al., showed an impaired migration function of EPCs in patients with coronary artery disease [83]. In hypertensive patients with left ventricular hypertrophy, the circulating levels and adhesive function of EPCs were lowered compared to non-hypertensive patients [84]. A significant reduction of EPCs number and proliferation rate has been also observed in patients with peripheral artery disease [85] alone and combined with diabetes [70,86].

### 4.3. Endothelial Progenitor Cells Dysfunction in Obesity

In obese patients, a decrease in EPCs number associated with significantly impaired clonogenic properties and an altered capacity to incorporate into tubule structures has been observed [87]; the decrease in EPCs number was reversed by weight loss [88,89]. In a C57BL/6J mice model of obesity induced by high fat diet, the number of EPCs from adipose tissue was significantly lower, as well as the circulating level of EPCs in response to ischemia, compared to control mice. The colony-forming capacity of peripheral blood-derived EPCs and the angiogenic capacity in response to ischemic stimulation were markedly altered in the obese mice compared to controls [90]. In addition, an impaired recovery of damaged endothelium, reduced EPCs angiogenesis ability and left ventricular ejection fraction, and an increased left ventricular remodeling have been observed in the obese compared to control mice [91].

### 4.4. Endothelial Progenitor Cells Dysfunction and Dyslipidemia

Dyslipidemia is characterized by increased triglyceride concentrations, decreased plasma high-density lipoprotein (HDL)-cholesterol levels and an increased proportion of small, dense low-density lipoprotein (LDL) particles, despite normal LDL-cholesterol. Hypercholesterolemia has been associated with reduced EPCs availability [92,93]. In vitro exposure of EPCs to oxidized-LDL decreased their number and impaired their adhesive, migratory and tube-formation capacities in a dose-dependent manner [94].

### 4.5. Endothelial Progenitor Cells Dysfunction and NAFLD

NAFLD is currently well recognized as a hepatic manifestation of MetS [95] and has been associated with obesity, insulin resistance, systemic inflammation and advanced atherosclerosis [96,97]. In addition, NAFLD has been related to endothelial dysfunction [98]. Patients with NAFLD have decreased number and function of circulating EPCs associated with features of MetS [99]. However, it has been shown that the number of EPCs was higher in patients with NAFLD and MetS in comparison to those without these conditions, and that the EPCs number was directly proportional to the degree of liver steatosis. The increase in EPCs number could be considered as a compensatory mechanism against endothelial injury [100].

## 5. Mechanisms Potentially Associated with Impaired Functionality of Endothelial Progenitor Cells

**Figure 1:**
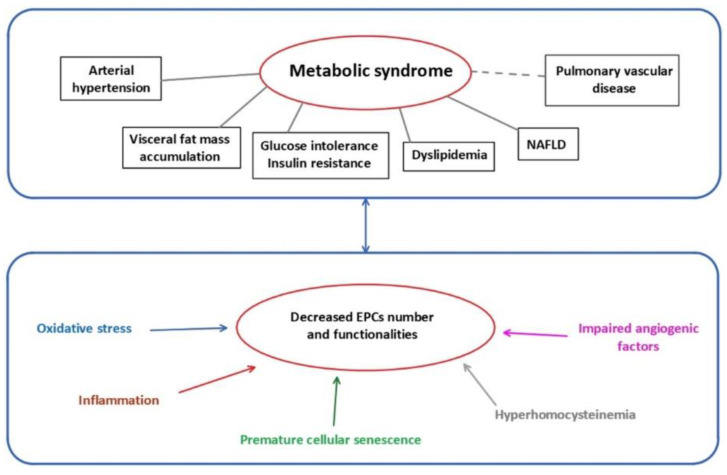
Metabolic syndrome and mechanisms related to EPCs dysfunctions. EPCs: endothelial progenitor cells; NAFLD: non-alcoholic fatty liver disease.

Several mechanisms have been identified to impair EPCs functionality (Figure 1).

### 5.1. Oxidative Stress

ROS are chemically reactive molecules formed during the metabolism of molecular oxygen. ROS are necessary in several biochemical processes such as intracellular signaling, cell differentiation, growth arrest, apoptosis, immunity and defense against microorganisms. The natural antioxidant system consists of a series of antioxidant enzymes, such as superoxide dismutases (SOD), catalase and glutathione peroxidase, and of endogenous antioxidant compounds. Oxidative stress occurs when the amount of ROS exceeds the antioxidant capacity. Excessive ROS production can interact with cellular macromolecules and then enhance the process of lipid peroxidation, cause DNA damage and/or induce protein and nucleic acid modifications [101], leading to decreased biological activity, dysregulated metabolism and alterations in cell signaling. In addition, oxidative stress can affect NO synthesis and bioavailability. Superoxide anion is known to interact with NO, leading to the formation of peroxynitrite, a highly reactive and toxic species able to modify macromolecules, such as lipids, proteins and DNA. Moreover, it has been suggested that increased oxidation of BH_4_ to 7,8-dihydropteridine (BH_2_) results in a reduced availability of this cofactor for eNOS [102], which impairs its activity and therefore NO production. Additionally, oxidative stress leads to increased endothelial-derived constricting factors such as endothelin-1 (ET-1), angiotensin II, thromboxane A2 and prostaglandin H2, thus enhancing vasoconstriction and contributing to endothelial dysfunction [103]. In addition, asymmetric dimethylarginine (ADMA), an endogenous competitive inhibitor of eNOS, inhibits the formation of NO and can lead, via eNOS uncoupling, to increased superoxide radical generation. Oxidative stress has been associated with decreased EPCs levels associated with reduced capability of mobilizing, migrating and incorporating into existing vasculature [62,104,105,106].

The expression of antioxidant enzymes, such as SOD, catalase and glutathione peroxidase, in EPCs is higher compared to endothelial cells [107] in the purpose to improve EPCs survival within the oxygen-poor environment of the bone marrow, as well as to support the ability of EPCs to engraft within ischemic tissues during the vasculogenesis process [107,108,109,110]. Dyslipidemia can lead to oxidative stress and so to EPCs dysfunction. In fact, oxidized-LDL, as oxygen donors, are responsible for inciting and perpetuating oxidative stress through a spiral of redox-based reactions, which impairs the vasculogenic function of EPCs [111]. In contrast, HDL, which have antioxidant and anti-inflammatory properties, have a positive impact on EPCs physiology [112].

Mitochondria play an important role in energy homeostasis by the production of ATP via oxidative phosphorylation and the oxidation of metabolites via the Krebs’s cycle and the beta-oxidation of fatty acids. Mitochondrial dysfunction is the major source of ROS production (0.2% to 2% of total oxygen taken up by cells), mainly at complex I (NADH CoQ reductase) and complex III (bc1 complex). Under normal conditions, the overproduction of ROS in mitochondria is restricted via enzymatic and non-enzymatic defense systems to protect cellular organelles from oxidative damage. However, when antioxidant defenses are overwhelmed, there is an overproduction of ROS, which then leads to oxidative damage to proteins, DNA and lipids in mitochondria [113,114]. An altered mitochondrial activity in EPCs has been observed in patients with cerebrovascular disorder [115] and type-2 diabetes [116].

### 5.2. Cellular Senescence

Cellular senescence is a biological phenomenon triggered by potentially harmful stimuli, during which the cell interrupts the division process, entering a state of cell cycle arrest and becoming quiescent. Senescence is a protective mechanism affecting the majority of the cells within an organism [117]. At the phenotypic level, senescent cells acquire a characteristic flattened and enlarged morphology with accumulation of lipofuscin, which is a marker of highly oxidized, insoluble proteins [118]. Cellular senescence is also characterized by a decline in the DNA replication in the cells, until they cease to proliferate, associated with molecular changes in elements related to the cell cycle (pRb, p21^WAF^, p16^INK4a^ and p53) [119]. Moreover, senescent cells undergo chromatin and secretome changes, genomic and epigenomic damage, unbalanced mitogenic signals and tumor-suppressor activation [120]. In addition to these common characteristics, replicative senescence can be identified by a decline in telomere length with each cell cycle [121]. Replicative senescence is an irreversible phenomenon, in contrast to stress-induced premature senescence (SIPS). SIPS is initiated in young cells via different mechanisms, such as oxidative stress, and has been associated with the over-expression of p16^INK4a^ and the decreased functionality of the anti-aging protein sirtuin-1 [122]. Moreover, senescent cells can exhibit an upregulation and secretion of growth factors, such as proinflammatory cytokines (IL-6 and IL-1), chemokines (IL-8, chemokine ligands family members), macrophage inflammatory protein and insulin-like growth factor, and also a release of extracellular matrix-degrading proteins (MMP family, serine proteases and fibronectin); the overall effect of these phenomena leads to the senescence-associated secretory phenotype (SASP) [123]. It is important to note that most senescent cells are resistant to some apoptosis signals, therefore they become senescent [119].

EPCs isolated from cord blood of diabetic mothers displayed in vitro premature senescence and impaired proliferation and in vivo reduced vasculogenic potential compared to uncomplicated pregnancies [124]. Oxidative stress and senescence are hallmarks often associated. An increased ROS production as well as oxidized-LDL have been associated with cellular senescence of EPCs [109,125], decreasing their number and impairing their function. Moreover, an association has been observed between oxidative DNA damage, decreased telomerase activity and decreased telomere length in EPCs isolated from patients with MetS and coronary artery disease [126].

### 5.3. Impaired Angiogenic Function

Angiogenesis is important to maintain the integrity of tissue perfusion, which is crucial for physiologic organ function. EPCs, and more particularly ECFCs, play a major role in the angiogenic process. In fact, it has been shown that ECFCs contribute not only to maintain microvasculature but also to stimulate postnatal angiogenesis [127,128,129].

NO is necessary for angiogenesis to occur [130]. NO is involved in the mobilization of EPCs and improves their migratory and proliferative activities [131], notably by the regulation of their angiogenic activity [132,133]. NO-mediated signaling pathways are essential for EPCs mobilization from the bone marrow [134,135,136]. NO regulates migration of EPCs into ischemic sites [61,136,137] and improves their survival [138]. A link between eNOS expression/functionality and EPCs function has been described [139]. In diabetic EPCs, eNOS activity is decreased, probably due to eNOS uncoupling, leading to reduced NO production and thus to decreased migratory capability, which is restored by exogenous NO administration [136,140,141]. The NO-donor sodium nitroprusside improved migration and tube formation, which was impaired by hyperglycemia [142]. ADMA levels are increased in diabetes [143] and it has been shown that ADMA decreased EPCs proliferation and differentiation, in a concentration-dependent manner [106].

NO can interact with angiogenic factors. Vascular endothelial growth factor (VEGF) plays an important role in EPCs differentiation and vascular repair [144,145]. A reciprocal relation between NO and VEGF has been demonstrated. The synthesis of VEGF can be induced by NO [146,147], and VEGF increases NO production by eNOS, promoting angiogenesis [148]. In patients with coronary heart disease, a reduced NO bioavailability has been observed, associated with altered VEGF expression and subsequently impaired EPCs functionality [149,150]. Moreover, a decreased secretion of NO and VEGF induced by hyperglycemia condition or advanced glycation end-products decreased activity of SOD, and so impaired EPCs function, such as migration and tube formation [151].

### 5.4. Inflammation-Induced EPCs Dysfunction

EPCs function and maturation are extremely sensitive to inflammation mediators. Autoimmune diseases like systemic lupus erythematosus (SLE) or rheumatoid arthritis as well as metabolic anomalies like type-2 diabetes mellitus demonstrate endothelial dysfunction related to chronic inflammation and later to CVD [152]. Patients with SLE displayed a chronic inflammatory state and a higher risk of MetS associated with a decreased level of circulating EPCs [153]. Interestingly, adipokines are critical mediators of inflammation and insulin resistance in SLE-associated MetS [154].

The level of type-I interferon impacts the EPCs number and function [155], especially in reducing their ability to repair vascular damage [156,157]. In a murine model, a type-1 interferon receptor knockout led to increased EPCs number and function, with improved neoangiogenesis and cell differentiation [158]. The blockade of IL-18 also enhanced differentiation of EPCs [159]. Increased expression of tumor necrosis factor α (TNFα) had detrimental effects on EPCs function as impaired proliferation, migration and tube formation [160]. Stromal cell-derived factor-1 (SDF-1) is a cytokine stimulating the recruitment of proinflammatory cells that contributes to EPCs mobilization [161]. In patients with type-2 diabetes, hyperglycemia reduces the level of VEGF and SDF-1 secretion from endothelial cells via the hypoxia-inducible factor/hypoxia-response element pathway and dipeptidyl peptidase-4 activity [162], therefore decreasing the mobilization of EPCs from bone marrow to circulation [163,164] and impairing the regulation of growth, migration and survival of EPCs [165].

### 5.5. Epigenetic Regulation

#### 5.5.1. MicroRNAs

MicroRNAs (miRNAs) have been identified to play important roles in the post-transcriptional regulation of gene expression influencing several cellular processes which contribute to disease [166,167]. Several miRNAs have been identified to regulate endothelial cell functions, such as cell proliferation, senescence, migration, differentiation and vascular tubule formation [168,169,170]. Therefore, an alteration of miRNAs expression can contribute to EPCs dysfunctions. It has been shown that miR-126 expression is necessary to downregulate Spred-1 to activate Ras/ERK/VEGF and PI3K/AKT/eNOS signaling pathways, improving EPCs proliferation and migration [171]. MiR-130a expression is involved in the proliferation, migration and colonies formation via RUNX3/ ERK/VEGF and PI3K/AKT signaling pathways [172]. Additionally, miR-31 is involved in the expression of several proteins implicated notably in the differentiation of bone-forming stem cells into mesenchymal and fat tissues [173,174]. In EPCs isolated from patients with type-2 diabetes, a downregulation of miR-126 and miR-130a, but an increase in miR-31 expression have been associated with an impairment of their function [171,172,175]. In addition, miR-34a overexpression led to increased EPCs senescence, associated with decreased sirtuin-1 functionality [176] and elevated miR-31-triggered apoptosis, which impaired EPCs functions in diabetic patients [177].

#### 5.5.2. DNA Methylation

DNA methylation is the best-known epigenetic mechanism, and usually leads to repressed transcription of the involved gene. Methylation takes place on CpG islands located mainly in the promoter region of the genes. Usually, if the CpG islands in the promoter region are unmethylated, the gene is transcribed, but when a significant part of these islands is methylated, the gene can no longer be transcribed, being so silenced. DNA methylation can be altered by early environmental factors [178]. Methyl CpG binding protein 2 (MeCP2) is an important member of the methyl-CpG binding protein family. It has been shown that overexpression of MeCP2 reduced angiogenesis, via decreased protein levels of p-eNOS/eNOS and VEGF and induced senescent EPCs dysfunction through sirtuin-1 promoter hypermethylation [179].

#### 5.5.3. Histone Modification

In the nucleus, DNA is packaged into chromatin as repeating units of nucleosomes, which form a “beads-on-a-string” structure that can compact into higher order structures to affect gene expression. Nucleosomes are composed of 146-bp DNA wrapped in histone octamers (composed of two H2A, H2B, H3 and H4) and are connected by a linker DNA, which can associate with histone H1 to form heterochromatin. Histone proteins contain a globular domain and an amino-terminal tail, with the latter being post-translationally modified. The post-translational modifications of lysine (acetylation, methylation, ubiquitination, sumoylation), arginine (methylation), as well as serine and threonine (phosphorylation) are the most described. It has been shown that the transcriptionally active H3K4me3 state leads to the activation of multiple pro-angiogenic signaling pathways (VEGFR, CXCR4, WNT, NOTCH, SHH) which improved the capacity of EPCs to form capillary-like networks in vitro and in vivo [180]. The concomitant inhibition of silencing histone modification (H3K27me3) and enhancement of activating histone modification (H3K4me3) improved eNOS expression in EPCs [181].

### 5.6. Hyperhomocysteinemia

Hyperhomocysteinemia is a clinical condition characterized by a high level of homocysteine in blood (above 15 µmol/L) [182,183,184]. Homocysteine is a sulfur-containing amino acid synthesized during the metabolism of methionine. It is catabolized either by remethylation to methionine, catalyzed by methionine synthase or betaine homocysteine methyl transferase, or by transsulfuration, catalyzed by cystathionine β-synthase (CBS) and cystathionine γ-lyase (CSE), leading to cysteine, a precursor of glutathione [185,186,187]. CBS and CSE require vitamin B6 as co-factor. A rate-limiting CBS enzyme as well as an insufficient dietary supply of cofactors have been involved in severe cases of hyperhomocysteinemia [188,189,190]. Other causes have also been identified, such as smoking, renal failure and other systemic diseases [191].

Hyperhomocysteinemia is an independent risk factor for cardiovascular disorders [192]. It has been associated with endothelial dysfunction in several animal models [193,194,195]. Hyperhomocysteinemia has been shown to contribute to endothelial dysfunction by induction of oxidative stress. In fact, hyperhomocysteinemia increases inducible nitric oxide synthase (iNOS) synthesis and ROS production [196], associated with SOD inactivation [197,198], so generating important quantities of peroxynitrite [196,199] and, therefore, contributing to nitrative stress which induces severe damage to proteins, lipids and DNA [200,201,202]. EPCs are particularly sensitive to oxidative stress, so hyperhomocysteinemia can contribute to EPCs dysfunction. It has been shown that homocysteine dose- and time-dependently impaired EPCs proliferative, migratory, adhesive and in vitro vasculogenesis capacities [203]. A significant decrease in circulating EPCs number and impaired functional capacity were observed in patients with hyperhomocysteinemia [204]. In mice, hyperhomocysteinemia-induced nitrative stress contributed directly to the injury of EPCs, by decreasing their survival rate, inducing apoptosis and necrosis [205]. In patients with stroke, high plasma level of homocysteine has been associated with a reduced number of EPCs colonies related to apoptosis [206,207], and administration of B vitamins (B6, B9) was able to attenuate such effects [206]. DNA methylation is an important process for gene transcription and therefore for regulation of protein expression. EPCs isolated from bone marrow in a mice model fed with a high methionine-rich diet displayed a reduced adhesion capacity and tube formation abilities associated with hyper-methylation in the CpG islands of the CBS promoter, leading to downregulated CBS expression. Such dysfunctions were reversed by administration of a DNA methylation inhibitor to mice fed with a high methionine-rich diet [208].

Homocysteine and hydrogen sulfide (H_2_S) are interconnected. H_2_S is an endogenous gasotransmitter, produced by CSE, CBS and 3-mercaptopyruvate sulfur transferase [187,209,210]. H_2_S acts via the S-sulfhydration of cysteine residues (-SH) of target proteins to form persulfide group (-SSH), which can modify the structure and activity of various target proteins [210,211]. H_2_S is emerging as an essential contributor to homeostasis of endothelial function, besides NO. It is involved in the regulation of several systems such as cardiovascular, nervous, gastrointestinal and renal systems, but also in the inflammatory and immune responses [212]. In the cardiovascular system, H_2_S is produced in cardiomyocytes, vascular endothelial cells, smooth muscle cells and EPCs [213]. H_2_S exerts antioxidant, anti-apoptotic, anti-inflammatory and vasoactive activities, and regulates proliferation, migration and angiogenesis, in an autocrine and paracrine manner [209,210,214,215,216]. H_2_S induces vasorelaxation by opening of K_ATP_ channels in vascular smooth muscle cells and partially through a K^+^ conductance in endothelial cells [217]. H_2_S can decrease inflammation by inhibiting transcription factors such as NF-kB [218]. H_2_S can reduce oxidative stress, through direct scavenging of oxygen and nitrogen species and enhancing antioxidant defense mechanisms, notably via the Keap1/Nrf2 pathway, and delays senescence [219]. In addition, H_2_S can also interact with the NO/NOS pathway to control vascular function, thanks the inhibition of phosphodiesterases in smooth muscle cells, by PI3K/AKT-dependent phosphorylation of eNOS in Ser1177 and by stabilization of eNOS in the dimeric state [220,221,222]. Moreover, H_2_S is a major factor to ensure EPCs functionality. In diabetic leptin receptor deficient db/db mice, the H_2_S plasma levels were significantly reduced and associated with impaired EPCs functionality such as tube formation, adhesive function and wound healing by decreasing angiogenesis process [223]. Shear stress was found to improve several EPCs functions, such as proliferation, migration, tube formation and reendothelialization [224,225,226], probably through enhancement of H_2_S production [227]. Indeed, shear stress was able to increase H_2_S production and CSE protein expression in human EPCs in a dose- and time-dependent manner, and to improve EPCs proliferation, migration and adhesion capacity [228].

As biogenesis of H_2_S and homocysteine is regulated by each other and imbalance between both molecules seems implicated in several cardiovascular disorders, the H_2_S/homocysteine ratio could be useful for cardiovascular risk prediction [186].

## 6. Developmental Programming of Cardiometabolic Diseases

The nutritional environment during the fetal and perinatal periods plays a major role not only in optimal offspring development, but also in adult health. In the 1980s, D. Barker observed, in a large cohort of adult men and women in Hertfordshire (UK), that a low birth weight (LBW), which is an indirect clinical marker of inappropriate intrauterine development, was inversely correlated with the risk of CVD and mortality [229]. Epidemiological studies on the Dutch Famine and on the Great Chinese Famine have reported an association between alterations in early nutrition and later development of cardiometabolic diseases [230,231]. These observations suggested the existence of a critical time window, from conception throughout pregnancy to early infancy, that is sensitive to long-lasting effects of environmental perturbations and could therefore potentially lead to the determination of final health outcomes by mis-programming. During this period of vulnerability, epigenetic modifications have been identified to play an important role in the regulation of the later development and long-term health outcomes.

LBW, as a consequence of intrauterine growth restriction (IUGR) or preterm birth, is associated not only with important perinatal mortality and morbidity, but also with long-term outcomes, such as cardiometabolic disorders [232]. Infants born following IUGR are at increased risk of elevated arterial blood pressure during infancy [233], adolescence [234,235], young adulthood [236] and later in life [237,238,239]. Among the mechanisms potentially involved in the development of arterial hypertension, alterations of the vascular system have been identified to play an important role, in addition to the long-term effects of decreased nephron endowment and hypothalamic-pituitary-adrenal axis hyperactivity [240]. Indeed, infants born following IUGR display notably impaired endothelium-dependent vasodilation and an increase in some markers related to arterial stiffness, such as increased intima media thickness and decreased arterial compliance [241,242,243,244]. Primary cultures of placental and umbilical endothelial cells derived from pregnancies complicated by IUGR showed abnormal phenotypes [245], characterized by an altered expression of proteins involved in NO-dependent vasodilation and changes in the proteome profile [246], suggesting an early programming of endothelial dysfunction.

In a rat model of IUGR induced by impaired maternal nutrition, Grandvuillemin et al. observed as early as at 5 weeks after birth, an impaired endothelium-dependent vasodilation in response to acetylcholine in the IUGR group, due to an upregulation of the arginase pathway and eNOS uncoupling, whereas no elevation in arterial blood pressure was observed at this age [247]. Thereafter, in adulthood, IUGR males displayed a higher arterial blood pressure, an increased vasoconstriction in response to angiotensin II, normalized by tempol (a radical scavenger and SOD-mimetic), an impaired endothelium-independent vasodilation in response to sodium nitroprusside in the aorta and a microvascular rarefaction as compared to control animals. These dysfunctions have been associated with increased vascular superoxide anion production mediated by NADPH oxidase and decreased cellular antioxidant glutathione levels in late gestation [248,249], so suggesting that nutrient depletion during fetal development could decrease antioxidant defenses, therefore leading to oxidative stress and vascular dysfunction.

Regarding metabolic alterations, a relationship has been mentioned between LBW and the development of type-2 diabetes in adulthood [250], obesity in 10- to 13-year-old schoolchildren [251] and the development of MetS in young adults [252]. Infants born following IUGR are also at risk to develop NAFLD later in life [253]. An association has been observed between LBW and increased levels of liver enzymes, such the aspartate aminotransferase (ASAT) and alanine aminotransferase (ALAT), measured in women aged 60–79 years, suggesting an impaired hepatic cellular function in these individuals [254].

Data have suggested that fetal life could alter the EPCs number and functionality. In fact, Meister et al. observed a 50% decrease in CD34^+^ cells in preterm neonates compared to term neonates [255]. In addition, a reduced number of circulating EPCs isolated from cord blood at birth has been observed in preterm infants [256], as well as in IUGR-complicated pregnancies [257,258]. EPCs isolated from cord blood of newborns with LBW displayed an impaired angiogenic function related to premature senescence, mediated by reduced sirtuin-1 functionality [122,259], and was associated with endothelial microparticle release due to the activation of the MKK6/p38MAPK pathways leading to the phosphorylation of Hsp27, which can contribute to the disruption of endothelial homeostasis [260]. In addition, neonatal EPCs isolated from diabetic mothers exhibited decreased proliferation and reduced vessel-forming capacity in vitro and in vivo, associated with premature senescence and oxidative DNA damage, compared with neonatal EPCs isolated from mothers with uncomplicated pregnancies [124].

Epidemiological studies and experimental models showed that impaired fetal growth induced short- and long-term adverse effects on lung structure and function [261,262,263]. In a rat model of IUGR induced by a maternal low-protein diet, lungs displayed a decreased alveolarization and vascularization [264]. In another rat model of IUGR induced by maternal undernutrition during pregnancy, adult exposure to chronic hypoxia induced pulmonary arterial hypertension (PAH) and pulmonary vascular remodeling correlated with molecular alterations in pulmonary vascular endothelial cells [265]. Impaired EPCs number and functional capacity were observed in patients with idiopathic PAH [266]. Circulating EPCs number was reduced in patients with PAH, which also exhibited abnormal levels of inflammatory mediators, cGMP, NO oxidation products and ADMA [267]. Although a correlation between MetS and pulmonary disease has not yet been clearly established, insulin resistance and dysregulated glucose metabolism have been found to be associated with the development of pulmonary vascular diseases such as PAH [268].

## 7. Reversibility of EPCs Dysfunction

**Figure 2:**
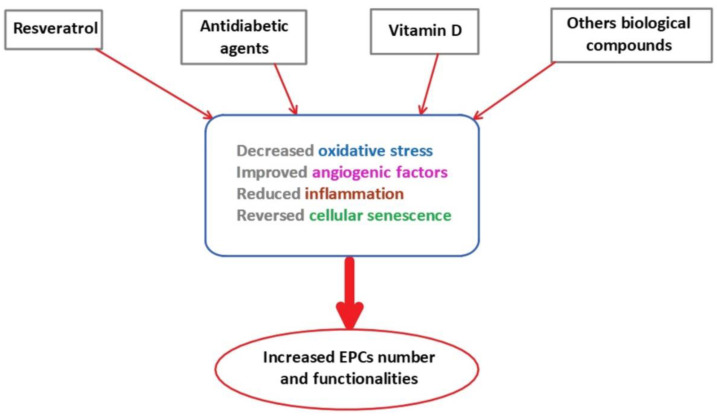
The effects of therapeutical agents on EPCs number and functionality. EPCs: endothelial progenitor cells.

Several compounds and lifestyle have been identified to improve EPCs functionality (Figure 2).

### 7.1. Resveratrol

Epidemiological studies have shown a low prevalence of CVD in the population of Southern France, despite a diet rich in saturated fat and cholesterol [269,270]. This so-called ‘French paradox’ has been attributed to a moderate consumption of red wine, in which resveratrol (trans-3,5,4’-trihydroxystilbene), a natural polyphenol, has been found in significant amounts. Resveratrol was first isolated in 1939 from Veratrum grandiflorum’s roots and is henceforth widely known as a phenolic compound with powerful antioxidant activity [271]. Resveratrol is present in several plants, including grape skins, grape seeds, giant knotweed, cassia seeds, passion fruit, white tea, plums and peanuts [272,273]. It has been shown that resveratrol can mimic calorie restriction and so promote good health [274]. Studies have reported that resveratrol can confer a protective effect on the cardiovascular system and metabolic disorders [275,276,277,278]. In fact, Wang et al. demonstrated that resveratrol promoted the proliferation, adhesion and migration of EPCs in a dose- and time-dependent manner and increased the expression of VEGF to further induce vasculogenesis [279,280], which was mediated by the activation of sirtuin-1 [281] and the regulation of the Synd4/AKT/eNOS pathway [282]. As a matter of fact, antioxidant properties of resveratrol are more likely attributed to its effect as regulator of the gene expression of pro-oxidative and antioxidative enzymes rather than its radical scavenging action [283]. Resveratrol also delayed the senescence of EPCs by increasing telomerase activity to maintain appropriate levels and function of EPCs [284,285], and by increasing sirtuin-1 functionality [122]. Resveratrol also prevented oxidative stress induced by diabetes in EPCs via sirtuin-1 activation [286] and modulation of the gene expression of SOD, glutathione peroxidase and NADPH oxidase subunit [287].

### 7.2. Oral Antidiabetic Agents

EPCs dysfunctions are closely related to impaired glucose metabolism and hyperglycemia. Therefore, improved hyperglycemia control could represent an interesting therapeutic target. Metformin is the main medication used in patients with type-2 diabetes. Metformin acts principally by inhibiting absorption of glucose in the gut, suppressing gluconeogenesis and glycogen synthesis, so improving the uptake and utilization of glucose, and also the sensitivity to insulin of peripheral tissues [288]. In addition, metformin exerts a positive effect on stem cells. In fact, this drug increased the number of circulating EPCs in patients with type-2 diabetes [289]. The potential effect of metformin on EPCs functionality could be due to its activation of AMP-activated protein kinase (AMPK) [290,291]. Indeed, it has been shown that AMPK stimulates eNOS and NO production [292], therefore improving EPCs mobilization and so their biological functions. In addition, AMPK activation by metformin can stimulate sirtuin-1 functionality [293], which was impaired by hyperglycemia, and so delay premature senescence [294]. It has been also shown that metformin improved angiogenesis of EPCs through increased VEGF-A levels and downregulated angiogenic inhibitors such as CXCL-10 and TIMP-1 [295].

Moreover, to potentially improve the positive effects of metformin on EPCs, it has been proposed to combine metformin with other oral antidiabetic compounds with antioxidant effects such as gliclazide [296,297,298]. It has been shown that this combination was more effective than metformin alone on the increase in the circulating EPCs level in patients with type-2 diabetes despite similar glycemic control [299]. Two other antidiabetic agents have been identified also to improve EPCs functions: the glucagon-like peptide-1 agonist and dipeptidyl peptidase-4 inhibitors. They act by increasing the incretin level to inhibit glucagon release and therefore improving the insulin secretion. It has been shown that glucagon-like peptide-1 agonist improved proliferation and differentiation of EPCs via upregulation of VEGF. The dipeptidyl peptidase-4 inhibitor sitagliptin [300] increased the circulating number of CD34^+^ cells and improved the adhesion ability of EPCs to the retinal vessels in Zucker diabetic fatty rats [301].

### 7.3. Vitamin D

Recently, it has been shown that the effects of vitamin D (1,25-dihydroxycolecalciferol) were not limited to the bone metabolism. In fact, the receptors for vitamin D (VDR) are present in several cell types, including endothelial cells. It has been suggested that vitamin D deficiency is a risk factor for the development of endothelial dysfunction [302]. Vitamin D supplementation can improve the endothelial function by regulating NO bioavailability, reducing oxidative stress and exerting anti-inflammatory effects [303]. However, the effects of vitamin D supplementation on EPCs functionality are relatively scarce. Supplementation with vitamin D increased the number of VDR on EPCs [304]. In patients with type-2 diabetes, vitamin D supplementation improved the colony forming capacity and the viability of EPCs. However, vitamin D supplementation seems to have no effect on EPCs angiogenesis markers expression in these patients [305].

### 7.4. Other Biological Therapies

#### 7.4.1. Supplementation of EPCs with Proangiogenic Factors

Pretreatment of EPCs with proangiogenic growth factors, such as VEGF, basic fibroblast growth factor and platelet-derived growth factor, and overexpression of eNOS were able to improve angiogenesis and wound healing in diabetic mice [306,307] and also displayed antiatherogenic properties [307]. In addition, injection of EPCs isolated from patients with peripheral arterial disease, supplemented with the soluble CD146 angiogenic factor, improved the generation of new blood vessels and restored blood flow in an animal model of hindlimb ischemia [308].

#### 7.4.2. The Renin–Angiotensin–Aldosterone System and Endothelin

The renin–angiotensin–aldosterone system (RAAS) and ET-1 represent two of the most potent vasopressor mechanisms [309,310] and so play a major role in the regulation of the cardiovascular system. RAAS and ET-1 are interconnected. In fact, angiotensin II can affect the synthesis of ET-1 which in turn regulates the RAAS [311]. Blockade of the RAAS with angiotensin converting enzyme inhibitors or angiotensin receptor blockers has been shown to increase EPCs number in patients with type-2 diabetes [312]. Using a combination of statin and angiotensin receptor blocker amplified the effect of angiotensin receptor blocker alone on the EPCs number in patient with type-2 diabetes [313,314].

Up-regulation of ET-1 has been shown to increase the circulating EPCs number in patients with myocardial infarction and type-2 diabetes [315], and a correlation has been observed between reduced EPCs number and reduced plasma level of ET-1 in children with arterial hypertension [316].

#### 7.4.3. Lifestyle Modifications

In diabetic mice, aerobic and resistance training increased PI3K and pAKT pathways, thus improving the proliferation and adherence capacities of EPCs [317]. In patients with MetS, exercise improved in vivo endothelial repair capacity of EPCs by increased NO production and reduced superoxide anion level [318]. Moreover, exercise combined to hypocaloric diet has led to weight reduction and so to increase in EPCs number and function in patients with CVD or type-2 diabetes [319].

#### 7.4.4. Treatment Using H_2_S Donors

It is important to maintain H_2_S plasma concentration at physiological level in the nanomolar order to control blood pressure, to correct endothelial dysfunction, vascular inflammation and redox state, and to improve neovascularization and wound healing. An ideal H_2_S donor should allow slow and gradual production and intracellular release of H_2_S. Several compounds have been developed. GYY4137 and AP39 are chemical agents developed as H_2_S donors, with slow realizing functions. Intravenous or intraperitoneal administration of GYY4137 improved vasodilation in aortic, renal and cardiac arteries in a rat model of Nω-nitro-L-arginine methyl ester hydrochloride (L-NAME)-induced hypertension [320]. GYY4137 also reduced atherosclerotic plaque formation and improved endothelium-dependent vasodilation by reducing vascular inflammation and oxidative stress in ApoE-/- mice [321]. AP39 reduced systemic blood pressure, heart rate and arterial stiffness in L-NAME treated rats [322]. Thanks the positive interaction and synergistic action between NO and H_2_S, a hybrid donor of NO and H_2_S has been synthesized, ZYZ-803, which improved blood flow and vascular density related to femoral artery ligation in mice [323]. Sodium hydrosulfide (NaHS) and 4-hydroxylthio-benzamide have been administrated as H_2_S diet supplementation in animal models and allowed to restore H_2_S level and EPCs functions notably by stimulating angiogenic pathways [214,215,223]. NaHS injection in a rodent model of carotid artery injury was able to promote reendothelialization following vascular injury by enhancing eNOS-dependent EPCs mobilization [324].

Oral compounds able to produce endogenous H_2_S have been developed. N-acetylcysteine, used to enhance cellular levels of glutathione, rapidly cleaved in vivo to yield cysteine, has been proposed. However, to date, there is no published data. The sulfur amino acid taurine was shown to increase the expression of CSE [325,326] and decrease blood pressure in patients with prehypertension.

Finally, natural compounds have been identified as a source of H_2_S, in particular polysulfides, such as garlic, which is considered as a natural medicine against hypertension. Polysulfides have been found to improve vasodilation properties, cardiac function and angiogenesis due to endogenous H_2_S release [32,33,34,35,36,37,38,39,40,41,42,43,44,45,46,47,48,49,50,51,52,53,54,55,56,57,58,59,60,61,62,63,64,65,66,67,68,69,70,71,72,73,74,75,76,77,78,79,80,81,82,83,84,85,86,87,88,89,90,91,92,93,94,95,96,97,98,99,100,101,102,103,104,105,106,107,108,109,110,111,112,113,114,115,116,117,118,119,120,121,122,123,124,125,126,127,128,129,130,131,132,133,134,135,136,137,138,139,140,141,142,143,144,145,146,147,148,149,150,151,152,153,154,155,156,157,158,159,160,161,162,163,164,165,166,167,168,169,170,171,172,173,174,175,176,177,178,179,180,181,182,183,184,185,186,187,188,189,190,191,192,193,194,195,196,197,198,199,200,201,202,203,204,205,206,207,208,209,210,211,212,213,214,215,216,217,218,219,220,221,222,223,224,225,226,227,228,229,230,231,232,233,234,235,236,237,238,239,240,241,242,243,244,245,246,247,248,249,250,251,252,253,254,255,256,257,258,259,260,261,262,263,264,265,266,267,268,269,270,271,272,273,274,275,276,277,278,279,280,281,282,283,284,285,286,287,288,289,290,291,292,293,294,295,296,297,298,299,300,301,302,303,304,305,306,307,308,309,310,311,312,313,314,315,316,317,318,319,320,321,322,323,324,325,326,327,328,329].

## 8. Conclusions

Patients with MetS-related cardiometabolic disorders, and particularly individuals born after IUGR, display impaired number and functionality of EPCs, characterized by alterations in their circulating level and their migration, proliferation and angiogenesis properties. These alterations are related to oxidative stress, cellular senescence, inflammation, impaired angiogenic factors, hyperhomocysteinemia, and are under the control of epigenetic mechanisms. However, it is not yet clearly established whether the dysfunction of EPCs precede or is a consequence of MetS.

Some therapeutical approaches have been proposed to reverse the EPCs dysfunction related to MetS. In the last few years, the use of stem cells has emerged as promising for regenerative medicine because of their capacities to contribute to organ repair and regeneration throughout life. In particular, the EPCs have been identified as having a clinical potential, notably in vascular regenerative applications [330,331] in ischemic diseases, such as myocardial infarction and peripheral vascular disease, but also in metabolic diseases, pulmonary and systemic hypertension [332,333].

A better understanding of the contribution of EPCs dysfunction in the developmental programming of cardiometabolic disorders could therefore help to design promising therapeutical approaches to prevent or reverse the development of some of the major non-communicable diseases at adulthood.
